# Jasmonic Acid-Mediated Antioxidant Defense Confers Chilling Tolerance in Okra (*Abelmoschus esculentus* L.)

**DOI:** 10.3390/plants14071100

**Published:** 2025-04-02

**Authors:** Weixia Liu, Jielin Wang, Dan Zhu, Xiaomin Yin, Gongfu Du, Yuling Qin, Zhiyuan Zhang, Ziji Liu

**Affiliations:** 1 Tropical Crops Genetic Resources Institute, Chinese Academy of Tropical Agricultural Sciences/Key Laboratory of Crop Gene Resources and Germplasm Enhancement in Southern China, Ministry of Agriculture and Rual Affairs/Key Laboratory of Tropical Crops Germplasm Resources Genetic Improvement and Innovation of Hainan Province, Haikou 571101, China; weixialiu@126.com (W.L.); zhudan@catas.cn (D.Z.); yinxm8303@126.com (X.Y.); dugongfu@catas.cn (G.D.); 2 Hainan Institute of Zhejiang University, Sanya 572025, China; 22216147@zju.edu.cn

**Keywords:** *Abelmoschus esculentus* L., chilling stress, JA, transcriptome, metabolome

## Abstract

Chilling stress inhibits the growth of okra (*Abelmoschus esculentus* L.), reduces its overall agricultural yield, and deteriorates fruit quality. Therefore, it is crucial to elucidate the mechanism through which okra plants respond to chilling stress. This study investigates the molecular mechanisms of chilling tolerance by comparing the transcriptome and metabolome of chilling-tolerant (Ae182) and chilling-sensitive (Ae171) okra varieties. We found that Ae182 exhibits higher antioxidant enzyme activities, including SOD, POD, CAT, and APX, suggesting it mitigates oxidative stress more effectively than Ae171. Metabolomics analysis revealed that Ae182 produces higher levels of jasmonic acid (JA) and JA-isoleucine (JA-Ile) under chilling stress, potentially activating genes that alleviate oxidative damage. Additionally, integrated analyses identified key transcription factors, such as AP2, BHLH, and MYB, associated with JA and chilling stress. These findings provide candidate genes for further research on chilling resistance in okra.

## 1. Introduction

Chilling stress is one of the major environmental factors that affect plant growth, development, and yield [1]. The fruit of okra (*Abelmoschus esculentus* L.) is rich in proteins, amino acids, vitamins, and dietary fiber. Additionally, okra contains oils, polysaccharides, pectin, and flavonoids, which offer various health benefits [2,3,4]. However, okra is highly sensitive to low temperatures. Its optimal growing temperature ranges between 25 °C and 30 °C, while an average temperature below 17 °C inhibits flowering and fruiting [5]. Temperatures below 14 °C result in stunted growth, significantly affecting both yield and quality, with the plant being able to tolerate a minimum temperature of only 8 °C [6]. Therefore, understanding the molecular mechanisms underlying okra’s response to chilling stress is essential for enhancing its chilling tolerance and improving cultivation efficiency.

Plants exhibit significant changes at multiple levels, including gene expression and metabolite accumulation in response to chilling stress. The primary damage caused by chilling stress is associated with the accumulation of reactive oxygen species (ROS), which induces oxidative damage. To mitigate oxidative stress, plants maintain ROS homeostasis through scavenging systems involving enzymes such as superoxide dismutase (SOD), peroxidase (POD), and catalase (CAT) [7]. Malondialdehyde (MDA) is a byproduct of lipid peroxidation and serves as a biomarker for oxidative stress. It indicates the degree of cell membrane damage caused by reactive oxygen species (ROS) [8]. Previous studies have demonstrated that chilling stress enhances the activity of antioxidant enzymes in the leaves of okra, promoting an increase in malondialdehyde (MDA) and soluble protein levels [9]. Chilling stress also disrupts cellular structures, leading to the accumulation of osmolytes, such as soluble sugars [10,11] and proline [12,13] in cabernet sauvignon grape, Citrus reticulata, Zea mays, and rice, which act as osmotic regulators. These osmolytes coordinate ROS homeostasis and maintain plasma membrane integrity, thereby reducing chilling-induced damage to the plant. In addition to causing oxidative stress, ROS functions as a signaling molecule that, in conjunction with plant hormones, regulates complex defense responses. It also interacts with other key signaling components, including nitric oxide (NO), calcium ions (Ca^2+^), kinases, phosphatases, and transcription factors, to mediate the C-repeat binding factor (CBF) pathway involved in chilling stress responses [7,14]. Additionally, exogenous NO treatment significantly increases the activities of SOD, POD, CAT, and ascorbate peroxidase (APX) in leaves, while inhibiting MDA accumulation, effectively mitigating oxidative damage and improving chilling tolerance in okra seedlings [15]. Abscisic acid (ABA) is regarded as a critical hormone in the response to chilling stress, as it promotes stomatal closure, regulates the expression of stress-responsive genes, stabilizes cell membrane structures, and controls osmotic stress tolerance by regulating the transcription of downstream genes [16]. Jasmonic acid (JA) plays a crucial role in enhancing plant tolerance to chilling stress by activating various signaling pathways that lead to the expression of cold-responsive genes. It modulates the ICE-CBF pathway, which is essential for the production of antifreeze proteins and other protective molecules. Additionally, JA helps in stabilizing cell membranes and enhancing antioxidant enzyme activity, thereby reducing oxidative damage caused by reactive oxygen species (ROS) [17,18]. Chilling stress induced the expression of JA biosynthesis-related genes and flavonol biosynthesis pathway [19]. These results demonstrated the importance of JA in the response to chilling stress. In okra seedlings, the exogenous application of low concentrations of salicylic acid (SA) under chilling stress has been shown to increase chlorophyll content, peroxidase activity, and proline levels while reducing catalase activity and MDA content, thereby enhancing chilling tolerance [20]. Additionally, exogenous NO treatment significantly increases the activities of SOD, POD, CAT, and ascorbate peroxidase (APX) in leaves, while inhibiting MDA accumulation, effectively mitigating oxidative damage and improving chilling tolerance in okra seedlings [15]. In most plants, the ICE-CBF-COR signaling pathway plays a central role in chilling stress responses [21,22]. CBF directly binds to the promoters of COR genes to induce their expression, thereby enhancing chilling tolerance. The expression of CBF is regulated by the ICE1 [18]. Additionally, CBF participates in regulating ROS scavenging, hormone metabolism, and the expression of genes involved in cellular protection [23].

Plant responses to chilling stress involve not only changes in gene expression but also the regulation of metabolites, including carbohydrates, lipids, amino acids, and secondary metabolites. The integration of multi-omics data provides comprehensive insights into gene regulatory networks and metabolic changes under chilling stress [24,25]. Amino acids may enhance chilling tolerance by activating polyamine synthesis pathways and the CBF-COR signaling pathway [26,27]. Carbohydrates such as starch and sucrose function as osmoprotectants, stabilizing cell membrane structures and reducing damage caused by chilling stress. They also participate in chilling stress responses through interaction with the ABA signaling pathway [28,29,30,31,32]. The metabolism of lipid compounds plays a crucial role in regulating chilling tolerance [33,34]. Secondary metabolites, including flavonoids, phenylpropanoids, and polyphenols, not only exhibit antioxidant activity but also contribute to chilling stress responses by modulating plant hormone signaling pathways [35,36,37,38,39,40,41].

However, the molecular mechanisms and key metabolic pathways underlying okra’s responses to chilling stress remain poorly understood. In previous study, two varieties, Ae171 (chilling-sensitive) and Ae182 (chilling-tolerant) were identified from approximately 200 okra germplasm resources collected domestically and internationally through systematic chilling stress treatment. The main purpose of this research was to provide a comprehensive analysis of physiological and biochemical indicators, transcriptomic and metabolomic data, and transcription factor networks between these two varieties under varying levels of chilling treatment. The results would reveal the physiological changes, gene expression patterns, and metabolic shifts involved in okra’s response to chilling stress, constructing a regulatory network for chilling tolerance.

## 2. Results

### 2.1. Phenotypic and Physiological Analyses of Chilling-Sensitive and Chilling-Tolerant Okra Varieties Under Chilling Stress

**Figure 1:**
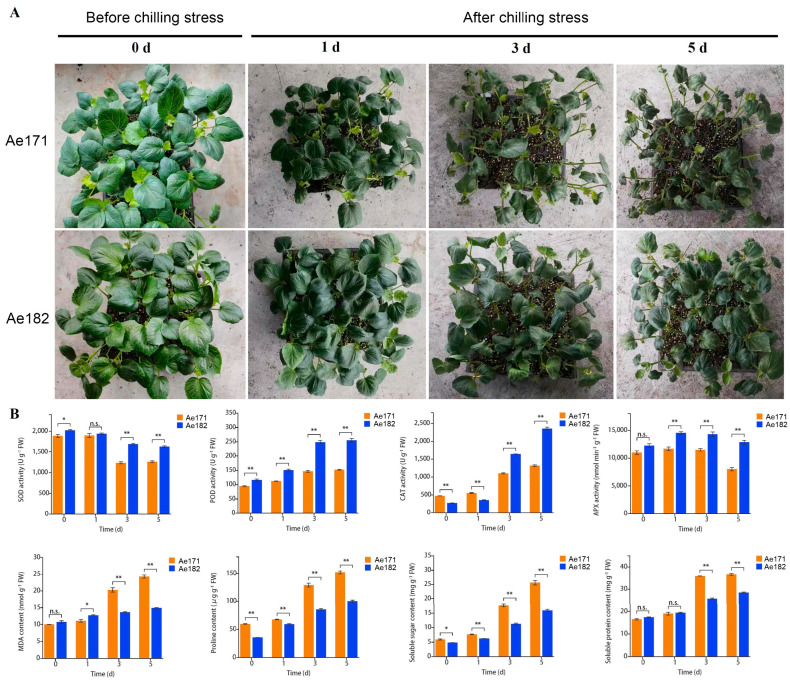
Comparison of the phenotype and physiological indicators of cold-sensitive (Ae171) and cold-tolerant (Ae182) okra varieties responding to cold stress at 0 days (CK), 1 day, 3 days, and 5 days after stress. (**A**) Phenotypic evaluation of Ae171 and Ae182. (**B**) Physiological indicators including POD activity, SOD activity, CAT activity, APX activity, MDA content, soluble sugar content, soluble protein content, and proline content. Error bars represent SD (n = 3). The statistical significance was determined via Student’s *t*-test, ** *p* ≤ 0.01 indicates a high level of significance, * *p* ≤ 0.05 indicates statistical significance, and n.s. denotes non-significant results.

The phenotypes and physiological indicators of AeAe171 (chilling-sensitive) and AeAe182 (chilling-tolerant) were analyzed under different durations of chilling stress. The results indicated that leaf wilting occurred later in AeAe182 compared to AeAe171. At the same treatment time points, Ae182 exhibited milder wilting symptoms than Ae171. After one day treated with chilling stress, wilting symptoms were already evident in Ae171, whereas Ae182 showed no obvious signs of stress. After three days, Ae171 displayed moderate wilting, while Ae182 exhibited only mild wilting. Ae171 showed severe wilting, with stem drooping, whereas Ae182 experienced moderate wilting but maintained upright stems after five days (Figure 1A). Compared with Ae171, the chilling-tolerant variety Ae182 demonstrated superior performance in several chilling-tolerance-related physiological indicators, while Ae171 performs well in some other physiological indicators (Figure 1B). For instance, after three and five days, the activities of key antioxidant enzymes, SOD, POD, CAT, and APX were significantly higher in Ae182 than in Ae171 (Figure 1B). MDA, a byproduct of lipid peroxidation and an indicator of oxidative stress, exhibited significantly higher levels in Ae171 than Ae182 after one, three, and five days, suggesting that Ae182 had a better capacity to mitigate oxidative stress under chilling conditions (Figure 1B). Additionally, after three and five days, the levels of osmoprotectants, including soluble sugars, soluble proteins, and proline were significantly higher in Ae171 than in Ae182 (Figure 1B). These results suggest that the superior chilling tolerance of Ae182 compared to Ae171 is mainly achieved through enhanced antioxidant defense enzyme activity rather than the accumulation of osmoprotectants.

### 2.2. Metabolomic Profiles of Ae171 and Ae182 in Response to Chilling Stress

**Figure 2:**
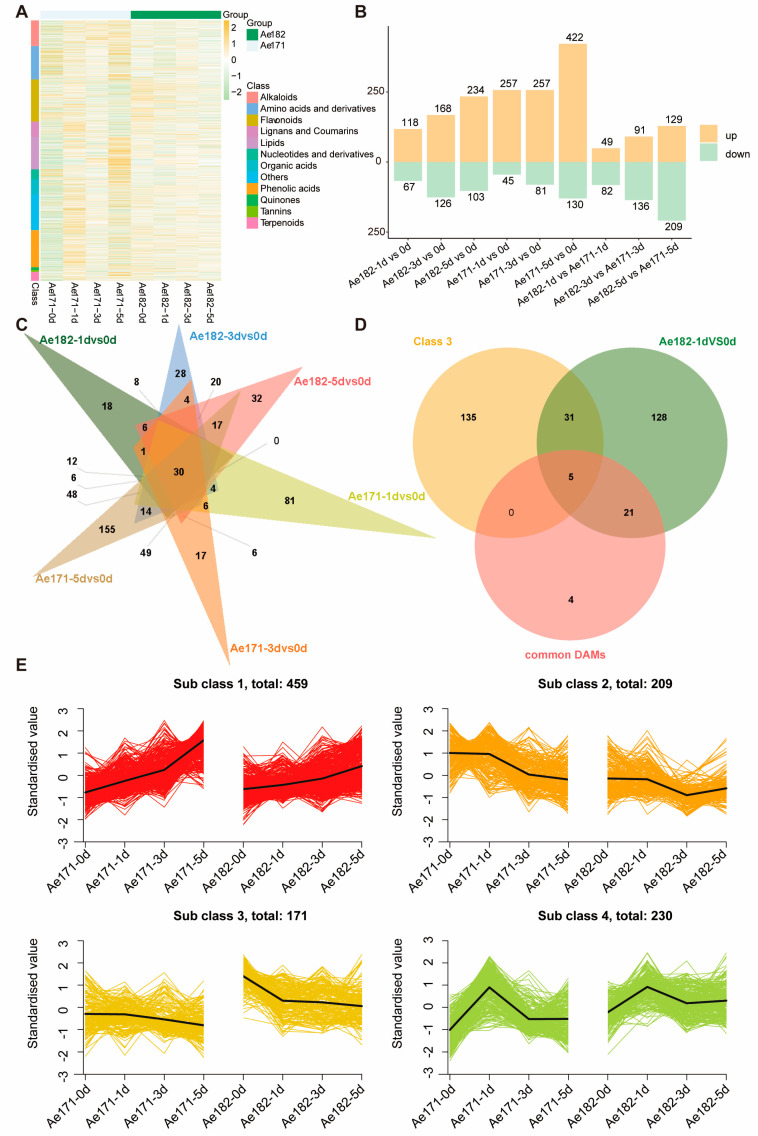
Metabolomic responses of okra Ae171 and Ae182 under chilling stress. (**A**) Hierarchically clustered heatmap of the 1687 metabolites from Ae171 and Ae182 after chilling treatment. Metabolites are categorized by class, as indicated by the color-coded sidebar. The color scale on the right indicates repression levels, with yellow representing high expression and green representing low levels. (**B**) Bar graph of upregulated and downregulated metabolites within and between okra varieties. (**C**) Venn diagram of the distribution of unique and shared DAMs among different comparisons between Ae171 and Ae182 at various time points. (**D**) Venn diagram analysis of common and specific metabolites within Ae171/Ae182, the differential metabolites of Ae182-1d vs. Ae171-1d, and the intersection of metabolites from Sub class 3. (**E**) k-means clustering of the 4 classes of metabolites, in which the number of metabolites within each class is shown. The x-axis depicts the time points during chilling stress, and the y-axis depicts the Z-score standardized per metabolite. Color lines represent the expression dynamics of each metabolite. Black lines indicate the representative expression for the cluster.

To explore the differences in the chilling-associated metabolic networks between Ae171 and Ae182 under chilling stress, leaves were sampled and subjected to an untargeted metabolic profiling method based on LC-MS/MS [42]. In total, 1687 metabolites were identified, including 166 alkaloids, 217 amino acids and derivatives, 273 flavonoids, 99 lignans and coumarins, 210 lipids, 64 nucleotide and derivatives, 98 organic acids, 240 phenolic acids,18 quinones, 4 terpenoids, 12 tannins, and 232 others (Figure 2A). By analyzing the metabolomic data, we identified differentially accumulated metabolites (DAMs) using a cutoff criterion of the variable importance in projection (VIP) > 1. Comparing the metabolite differences between the two okra varieties, the chilling-sensitive variety Ae171 exhibited more pronounced changes under chilling stress, with a sharp increase in upregulated metabolites, while the chilling-tolerant variety Ae182 showed a more gradual increase in upregulated metabolites. Additionally, Ae182 predominantly displayed downregulated metabolites compared to Ae171 (Figure 2B).

Based on the KEGG pathway analysis comparing Ae182 at 1 d versus 0 d under chilling stress, our study reveals significant alterations in key metabolic pathways that contribute to enhanced cold tolerance in okra plants. At 1 d post-treatment, we observed substantial upregulation of aminoacyl-tRNA biosynthesis and ABC transporter pathways, indicative of an enhanced capacity for protein synthesis and cellular material transport. The activation of glucosinolate biosynthesis pathways suggests an immediate reinforcement of defense mechanisms against cold-induced stress. Furthermore, the upregulation of purine and nucleotide metabolism pathways, along with amino acid biosynthesis pathways, demonstrates an increased turnover of nucleic acids and proteins, reflecting the plant’s initial metabolic adjustment to low-temperature stress. At 3 d after chilling exposure, consistent upregulation of ABC transporters and glucosinolate biosynthesis pathways was found, maintaining enhanced transport activity and defensive capabilities. The sustained elevation of aminoacyl-tRNA biosynthesis and various plant secondary metabolite pathways indicates continued protein synthesis and secondary metabolite production. Significant activity in nucleotide and purine metabolism pathways reflects ongoing nucleic acid synthesis. Notably, the upregulation of pathways related to amino acid biosynthesis and metabolism, particularly phenylalanine, tyrosine, and tryptophan biosynthesis, highlights the plant’s developing adaptive mechanisms in response to prolonged cold stress. By 5 d after chilling treatment, our results demonstrated persistent and significant upregulation of glucosinolate biosynthesis and ABC transporter pathways, confirming the maintenance of enhanced defense mechanisms and transport processes. The increased activity in aminoacyl-tRNA biosynthesis and starch and sucrose metabolism pathways suggests continued protein synthesis and energy management. The upregulation of nucleotide and purine metabolism pathways indicates sustained nucleic acid synthesis. Furthermore, the activation of specific amino acid metabolism pathways, particularly alanine, aspartate, and glutamate metabolism, reveals the plant’s progressive adaptation and increasing resilience to extended cold stress conditions (Supplementary Figure S1).

Comparative analysis of Ae182 and Ae171 at 1-day post-cold stress reveals significant upregulation of key metabolic pathways associated with enhanced cold tolerance in okra. Notably, the flavonoid and anthocyanin biosynthesis pathways demonstrate marked activation, suggesting strengthened antioxidant mechanisms and cellular protection against cold-induced oxidative stress. Concurrently, the upregulation of lipid metabolism pathways, including ether lipid and glycerophospholipid metabolism, indicates improved membrane stability and structural integrity. Furthermore, the enhanced phosphatidylinositol signaling system and plant hormone signal transduction pathways suggest a robust molecular response to chilling stress, collectively contributing to the observed cold tolerance in okra varieties. Three-day cold stress analysis reveals distinct metabolic adaptations in cold-tolerant okra through enrichment analysis of differential metabolites. Flavonoid biosynthesis emerges as the most significantly enriched pathway, underscoring its pivotal role in antioxidant activity and cellular protection. Sphingolipid metabolism shows notable enrichment, highlighting its dual function in maintaining membrane stability and facilitating signal transduction. Metabolic alterations in arginine and proline pathways potentially contribute to osmotic regulation and antioxidant defense mechanisms. The upregulation of anthocyanin biosynthesis further reinforces the plant’s antioxidant capacity, while alpha-linolenic acid metabolism suggests its involvement in modulating membrane fluidity and signaling molecule synthesis. The comprehensive enrichment of secondary metabolite biosynthesis pathways demonstrates their extensive role in stress response, with glutathione metabolism maintaining its essential function in antioxidant defense, albeit with lower enrichment levels. These metabolic adjustments collectively enhance the antioxidant capacity, improve membrane stability, and optimize osmotic regulation in cold-tolerant okra under chilling stress conditions. The 5-day cold stress analysis reveals continuing metabolic adaptations in cold-tolerant okra, with flavonoid biosynthesis maintaining significant enrichment, emphasizing its persistent role in antioxidant defense mechanisms. Pathways associated with alpha-linolenic acid metabolism and linoleic acid metabolism show prominent enrichment, indicating their ongoing importance in maintaining membrane fluidity and structural integrity under prolonged stress conditions. The notable enrichment of secondary metabolite biosynthesis and cofactor-related pathways reflects a comprehensive adaptive response to extended chilling stress. These findings illustrate the complex and sustained metabolic adjustments that contribute to the resilience of cold-tolerant okra varieties under prolonged cold stress conditions (Supplementary Figure S2).

To explore the common metabolite chilling resistance mechanisms in okra, a Venn diagram of differential metabolites within the two varieties revealed 30 common metabolites, including jasmonic acid (JA) and jasmonoyl-L-isoleucine, known for their contribution to plant chilling resistance (Figure 2C, Supplementary Table S1).

To gain a deeper understanding of metabolic alterations during chilling stress, 1682 metabolites were categorized into four distinct classes using the k-means clustering algorithm (Figure 2E). In Classes 1, 2, and 4, the clustering modules of metabolites in Ae171 and Ae182 exhibit nearly consistent trends. However, in Class 3, following exposure to chilling damage, Ae182 shows significant downregulation of certain metabolites compared to Ae171. These downregulated metabolites may be potential contributors to the cold tolerance observed in Ae182. Within the classes, there are 51 flavonoids, 23 phenolic acids, 19 other metabolites, and 16 lignans and coumarins, which collectively account for over 60% of the total 172 metabolites (Supplementary Table S2).

Furthermore, by examining the differential metabolites within Class 3 alongside metabolites from both okra varieties and those at one day (Figure 2D), five common metabolites emerged: 2-Dodecenedioic acid, jasmonic acid, jasmonoyl-L-Isoleucine, Phe-Glu-Leu, and glycoric acid, representing amino acids and derivatives, organic acids, lipids, and others. Notably, jasmonoyl-L-isoleucine, as the primary active precursor of jasmonic acid, underscores its significance in the context of chilling stress response in plants [43].

### 2.3. Ae171 and Ae182 Show Distinct Transcriptomic Alterations in Response to Chilling Stress

**Figure 3:**
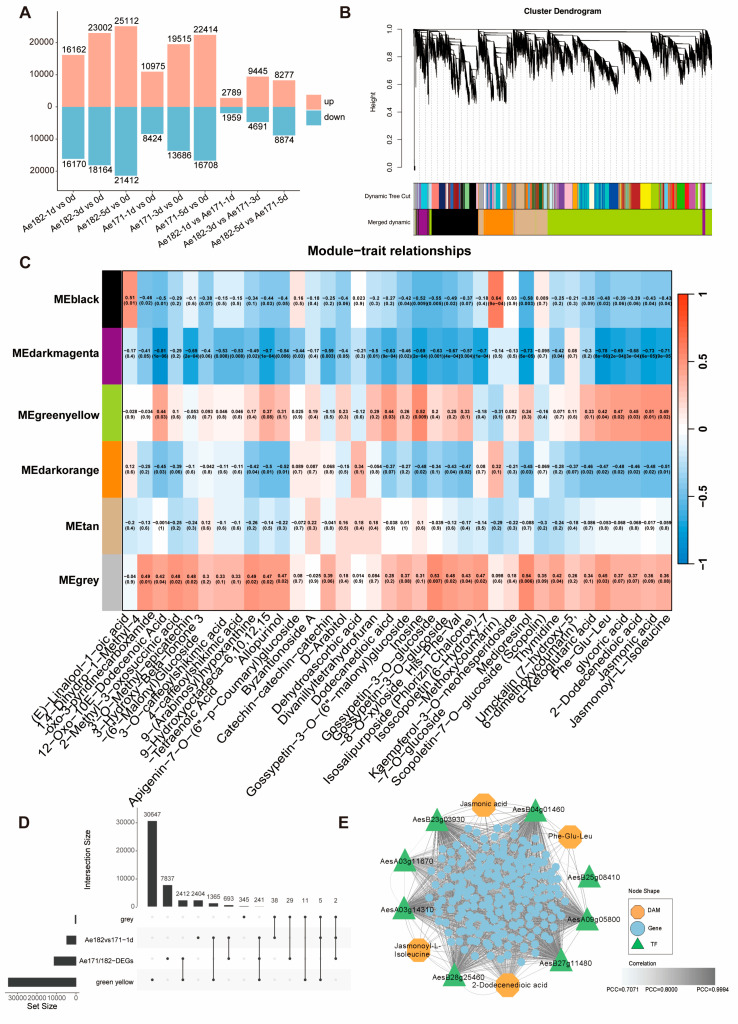
Transcriptomic alterations in Ae171 and Ae182 under chilling stress. (**A**) Bar graph of upregulated and downregulated metabolites within and between okra varieties. (**B**) Cluster dendrogram illustrating the hierarchical clustering of metabolites based on their expression profiles. The top section shows the dendrogram structure, indicating the similarity between clusters. Below, the dynamic tree cut is visualized, with distinct colors representing different modules. The merged dynamic tree cut highlights consolidated clusters, providing insight into the relationships among metabolite groups. (**C**) Heatmap illustrating module-trait correlations. Modules are labeled on the left, with distinct colors representing different groups of genes. Each column corresponds to a chilling-related metabolite. Red color indicates a positive correlation between the cluster and the metabolite. Blue color indicates a negative correlation. Numeric values within the cells denote the correlation coefficients, providing insight into how each module is associated with different metabolites. (**D**) Advanced Venn diagram (Upset) of the metabolome in different blocks. The total number of DAMs in each block is shown on the left. Upset plot illustrating the intersections among different gene sets in the analysis. Each vertical bar represents the size of the intersection between selected sets, while horizontal bars indicate the total size of each set. The sets include Ae182vs171-1d, Ae171vs182-DEGs, and MEgreen yellow from WGCNA, with specific intersections highlighted by connected dots below the bars. (**E**) Correlation network diagram depicting the interactions between differentially accumulated metabolites (DAMs), genes, and transcription factors (TFs). Nodes are shaped according to their type: circles for DAMs, triangles for genes, and squares for TFs. Edges represent interactions or associations, with the color gradient of the nodes indicating interaction strength—darker shades signify stronger associations. Line colors represent Pearson correlations, ranging from white (0) to dark gray, highlighting the correlation intensity.

To investigate the molecular basis of metabolic adaptation in response to chilling stress in Ae171 and Ae182, RNA-Seq data were generated using the samples for metabolome analysis. Illumina HiSeq RNA-sequencing yielded an average of 45.4 Gb and 34.6 Gb of high-quality reads for the Ae171 and Ae182, respectively. The average mapping of high-quality sequences was 92.94% for Ae171 and 95.37% for Ae182 against the *Abelmoschus esculentus* genome [44]. Differentially expressed genes (DEGs) were then analyzed in chilling-treated samples of both species to gain further insights into the transcriptional changes during chilling treatment. A total of 32,332 (16,162 upregulated and 16,170 downregulated), 41,116 (23,002 upregulated and 18,164 downregulated), and 46,524 (25,112 upregulated and 21,412 downregulated) DEGs were identified in Ae182 after 1, 3, and 5 d of chilling treatment, compared to 0 d. Comparison implying that a total of 19,399 (10,975 upregulated and 8424 downregulated), 33,201 (19,515 upregulated and 13,686 downregulated), and 39,122 (22,414 upregulated and 16,708 downregulated) DEGs were identified in Ae171 after 1, 3, and 5 d of chilling treatment, respectively (Figure 3A, Supplementary Table S3). Compared to Ae171, the transcriptome response of Ae182 to chilling stress is faster, with a greater number of upregulated genes.

To explore the common response mechanisms under cold stress, we conducted KEGG and GO analyses on the upregulated and downregulated gene sets shared by both types of okra. The KEGG results indicate that the upregulated genes are primarily enriched in the ethylene activation signaling pathway, jasmonic acid-mediated signaling pathway, and responses to bacterial-derived molecules, which suggests that these pathways play significant roles in the defense mechanisms against cold stress. Additionally, the GO enrichment of amino acid and fatty acid metabolism pathways highlights the metabolic adaptability of the plant under cold conditions. Conversely, the downregulated genes are mainly associated with flavonoid glycoside transferase activity and hormone-binding functions in the GO analysis, which may lead to reduced antioxidant capacity and affect the plant’s ability to respond to environmental changes (Supplementary Figure S3).

### 2.4. Key Gene Response to Chilling Stress Was Identified Using WGCNA in Orka

To study the relationship between these differentially expressed genes and metabolites, weighted gene co-expression network analysis (WGCNA) was subsequently undertaken to understand the regulatory mechanisms involved in metabolic alterations under chilling stress and explore co-expression networks of the DEGs (Figure 3B). To elucidate the common and unique cold-tolerant mechanisms in both non-cold-tolerant and cold-tolerant okra, a total of 36 metabolites were selected for comprehensive analysis. This selection comprises 31 metabolites derived from Class 3, with a specific focus on the comparison of Ae182 at 1 d versus 0 d, as well as five common metabolites identified across the three intersections, which were designated as candidate metabolites associated with WGCNA. Among these metabolites, there are eight types of flavonoids and five types of lipids. Additionally, this metabolite module includes jasmonic acid (JA) and its active precursors jasmonoyl-L-isoleucine, along with dehydroascorbic acid and other antioxidant or bioactive substances, which are critical for modulating oxidative stress and enhancing cold tolerance. Jasmonic acid (JA) has emerged as a pivotal signaling molecule in mediating plant responses to chilling stress, demonstrating its essential role in enhancing chilling tolerance through the regulation of stress-responsive gene expression. Previous research has revealed that JA orchestrates the activation of specific transcription factors, thereby inducing the upregulation of genes associated with chilling acclimation and stress defense mechanisms. In our investigation, particular attention was directed towards MEgreenyellow, a key component that exhibits significant associations with both JA and its biologically active conjugate, jasmonoyl-L-isoleucine (JA-Ile). Notably, MEgreenyellow represents approximately half of the potential metabolites identified in this context, highlighting its importance in the chilling stress response pathway (Figure 3C). To systematically identify the key transcription factors involved in the early response to chilling stress in chilling-tolerant plants, we performed an integrative analysis by intersecting the differentially expressed genes (DEGs) from the MEgreenyellow module with those identified in the Ae182 vs. Ae171-1d comparison. Through comprehensive annotation of the overlapping gene set, we successfully pinpointed potential regulatory transcription factors that may play pivotal roles in the initial chilling stress response (Figure 3D). This analysis resulted in a total of 241 unique genes. Ultimately, we discovered eight transcription factors, including two from the TCP family, two belonging to MYB family, and one each from the HLH family, AP2 family, bZIP_1 family and an unknown category (Supplementary Table S4). Subsequently, we mainly constructed a correlation network diagram of common metabolites, transcription factors, and genes within the module. There is a strong correlation between metabolites and genes, showing that these genes may play an important role in chilling tolerance of okra (Figure 2E and Supplementary Table S5).

## 3. Discussion

Chilling stress is an abiotic stress that adversely affects plant growth and agricultural yields [45]. Chilling stress typically restricts plant growth and development by interfering with the stability of proteins or protein complexes and reduces enzyme activity, such as the activity of ROS scavenging enzymes, which leads to the excessive production of reactive oxygen species (ROS) and oxidative stress [46]. These processe lead to photoinhibition and damage to photosynthesis, as well as significant membrane damage [47,48]. In our results, we found that SOD, POD, CAT, and APX activities are higher in Ae182 than in Ae171, which enables Ae182 to scavenge more reactive oxygen species (ROS), indicating that the okra-resistant chilling stress via alleviating oxidative stress response.

To further understand the mechanisms behind this resilience, we conducted comprehensive transcriptomic and metabolomic analyses. These analyses aimed to uncover the molecular and biochemical pathways that contribute to Ae182’s enhanced tolerance to chilling stress, providing deeper insights into its adaptive responses.

The transcriptomic analysis highlighted the enrichment of the ethylene and jasmonic acid signaling pathways among upregulated genes, indicating their crucial roles in defense strategies. Alterations in amino acids and fatty acid metabolism suggest enhanced cell membrane stability and functionality through protective compound synthesis. However, the downregulation of antioxidant-related genes and hormone-binding genes may reveal vulnerabilities in okra’s cold stress response, highlighting targets for improving cold tolerance. Future research should aim to functionally validate these key genes to develop effective strategies for enhancing okra’s cold resistance.

Metabolomic findings illustrated distinct responses between Ae171 (chilling-sensitive) and Ae182 (chilling-tolerant) to low temperatures. Ae171 showed more pronounced changes early in the stress response, indicating a reactive approach. In contrast, Ae182 demonstrated a more moderated and regulated response, characteristic of a plant better equipped to handle stress over time. This suggests Ae182’s chilling tolerance may stem from its ability to moderate stress mechanisms and efficiently activate protective pathways.

In metabolite-related analysis, we found that JA and its synthetic precursors exist at the intersection of various analyses, which attracts us to explore the role of JA in the cold tolerance of okra. Plant hormones are involved in mediating cold stress signaling [49,50]. When plants are exposed to cold stress, the levels of ABA and JA increase, while the levels of cytokinins, ethylene, and GA decrease. In chilling stress, JA play an indispensable role via activating the gene expression to attenuates chilling-induced oxidative stress [51,52]. Our findings particularly highlight the pivotal role of jasmonic acid (JA) in okra’s cold tolerance mechanism. The increased production of JA and JA-Ile in chilling-tolerant varieties underscores the importance of this hormone in attenuating chilling-induced oxidative stress.

Through integrated transcriptomic and metabolomic analysis, we identified many differentially expressed genes that may play important roles in okra’s cold tolerance. Among the investigated genes, we identified several transcription factors, including AP2 family members (TINY2, ERF114, and ERF115), MYB family members (GT2 and ASIL2), and the bHLH transcription factor PRE5, all of which are hypothesized to play critical roles in the chilling tolerance of okra. Specifically, TINY2 has been shown to exhibit significant activation under drought, cold, and ethylene treatments, with slight induction by methylasmonate (MeJA). ERF114 is induced by MeJA in a process dependent on COI1 and MYC2, forming a positive feedback loop that enhances the transduction of JA signaling. Additionally, the atypical JAZ protein JAZ8 interacts with ERF114 and ERF115, further implicating these transcription factors in the integration of JA and ethylene signaling pathways. Furthermore, we identified two TCP transcription factors, TCP3 and TCP19, whose roles in chilling stress are suggested by previous studies demonstrating that certain TCP members, such as CaTCP8, are cold-induced [53,54]. Collectively, these findings highlight the potential involvement of AP2, MYB, bHLH, and TCP transcription factors in mediating okra’s response to low-temperature stress, providing valuable insights into the molecular mechanisms underlying chilling tolerance.

In summary, our study highlights the complex interplay of biochemical and molecular pathways contributing to Ae182’s enhanced chilling tolerance. By understanding these mechanisms, we can develop targeted strategies to improve cold resistance in okra and other crops, ultimately enhancing agricultural productivity under stress conditions. Future research directions should focus on functional validation of the identified transcription factors and their specific roles in chilling tolerance. Particular attention should be given to the regulatory networks involving JA signaling and the interplay between different hormone pathways. Additionally, the potential role of TCP transcription factors in cold stress responses warrants further investigation, potentially opening new avenues for enhancing cold tolerance in okra and related species.

## 4. Materials and Methods

### 4.1. Experimental Materials and Chilling Stress Treatment

Two okra varieties, the chilling-tolerant okra “Lv Ru Yi” (Ae182) and chilling-sensitive okra “Fu Xing 434” (Ae171), were obtained from the Institute of Tropical Crop Germplasm, Chinese Academy of Tropical Agricultural Sciences, Haikou, China. Uniform and plump okra seeds were selected and sterilized with a 3% sodium hypochlorite solution for 10 min, followed by thorough rinsing with distilled water. The treated seeds were soaked until germination, after which they were sown into 50-cell seedling trays containing growth substrate until the bud length reaches about 0.2 cm. The seedlings were cultivated in the greenhouse, and after emergence, thinning was carried out to retain one seedling per cell. Twelve trays were prepared for each variety. After 35 days of growth, when the seedlings reached the three-leaf stage, they were transferred to a growth chamber for chilling stress treatment. The temperature was set to 10 °C/5 °C (day/night) with a 14 h photoperiod and 10 h dark period. One CK (0 day) and three treatment durations were applied: 1 day, 3 days, and 5 days. Each sample was collected from the second true leaf at 2:00 p.m. for physiological measurements and transcriptomic and metabolomic analyses. Three biological replicates for each treatment were performed.

### 4.2. Physiological Measurements

For sample preparation, fresh leaf tissues (0.5 g) were collected and immediately frozen in liquid nitrogen to preserve biochemical integrity. The samples were then ground into a fine powder using a pre-chilled mortar and pestle under liquid nitrogen. The powdered samples were homogenized in 5 mL of ice-cold extraction buffer (specific to each assay, as per the kit instructions) and centrifuged at 8000× *g* for 10 min at 4 °C. The resulting supernatants were carefully collected and used for subsequent analyses.

The following physiological indicators were measured for both varieties under the four chilling stress treatments: Superoxide dismutase (SOD) activity (WST-8 method; catalog no.: SOD-1-W), Peroxidase (POD) activity (catalog no.: POD-1-Y), Catalase (CAT) activity (catalog no.: CAT-1-Y), Ascorbate peroxidase (APX) activity (catalog no.: APX-2-W), Malondialdehyde (MDA) content (catalog no.: MDA-1-Y), soluble sugar content (catalog no.: KT-1-Y), soluble protein content (catalog no.: BCAP-1-W) and proline content (catalog no.: PRO-1-Y). All measurements were performed using commercial kits supplied by Keming Biotechnology Co., Ltd. (Suzhou, China).

### 4.3. Transcriptome Analysis

Okra leaves samples from four periods were used for transcriptome analysis, and three biological replicates were included for each group. A Trizol reagent was used to extract RNA from fresh leaves samples (Thermo Fisher Scientific, Waltham, MA, USA). followed by sequencing. The raw data were obtained by transcriptome sequencing on an Illumina NovaSeq 6000 platform (Illumina, San Diego, CA, USA), according to the manufacturer’s protocol. Clean data were obtained after filtering using a Trimmomatic algorithm. Mapping data were obtained by sequence alignment with the *Abelmoschus esculentus* genome [44] using HISAT2 2.1.0 [55]. The reads were assembled into transcripts using StringTie v2.1.7. Fragments per kilobase per million total mapped fragments (FPKM) were used to measure transcript levels. According to the gene expression of different sample groups, the DEGs were annotated and enriched. DESeq2 was used to analyze the differences in expression between sample groups in Rstudio (R4.4.1). Screening conditions for differentially expressed genes (DEGs) were |log2foldchang| ≥ 1 and *p*.adj < 0.05.

### 4.4. Metabolome Analysis

Four stages of okra leaves were sampled for metabolomic analysis by Metware Biotechnology Co., Ltd. (Wuhan, China), with each group comprising three biological replicates. Samples underwent vacuum freeze-drying followed by HPLC–MS analysis. Using an in-house database, metabolites were identified through their secondary spectral information and quantified using a multi-reaction monitoring (MRM) model. Standardized data processing was conducted for subsequent analysis. Principal component analysis (PCA) was employed to preliminarily assess the overall metabolic differences and variability among the sample groups. The square of the Spearman rank correlation coefficient (R2) served as the evaluation index for biological replicate correlation. Differences were calculated and compared based on grouping information, and a *t*-test was applied to determine the significance (*p* value) of each metabolite. The variable importance in projection (VIP) values were calculated through multiple cross-validation within the OPLS-DA model, combining fold change, *p* value, and VIP value to identify differential accumulated metabolites (DAMs). Differentially accumulated metabolites (DAMs) were identified based on the criteria of VIP ≥ 1 and fold change > 2 or <0.5. Mfuzz package in RStudio was used to cluster and visualize time-series metabolites accumulations data derived from metabolome analyses using k-means clustering.

### 4.5. Combined Transcriptome and Metabolome Analysis

To identify genes involved in the regulation of chilling tolerance in okra, we conducted a weighted gene co-expression network analysis (WGCNA) [56] on the substrates and transcriptomic data obtained from the chilling tolerance DAMs in okra. We performed a comparative analysis between Ae171 and Ae182, both within and between species. We performed a comparative analysis within two types of okra at 1, 3, and 5 days versus 0 days and between the two okras at 1 day to identify differentially expressed genes. Additionally, we analyzed the temporal metabolomic data to identify metabolites within Sub Class3 (C3) and those associated with the MEgreenyellow module in WGCNA that correlated with jasmonic acid and jasmonoyl-L-isoleucine.

We intersected the genes from these analyses to obtain a set of 241 genes, which were then annotated using pfam, COG, KOG, GO, and KEGG databases via the eggNOG-mapper [57,58,59,60,61]. This process led to the identification of 8 transcription factors. To explore the relationship between these transcription factors and the metabolites involved in chilling tolerance, we calculated Pearson correlation coefficients and visualized the resulting network using Cytoscape v3.9.1 [62].

## Data Availability

The original contributions presented in the study are included in the article/Supplementary Material. Further inquiries can be directed to the corresponding author. The raw RNA-seq data generated from this study have been submitted to NCBI SRA (https://www.ncbi.nlm.nih.gov/sra) under the accession number PRJNA1205086, accessed on 1 June 2025.

## Supplementary Materials

The following supporting information can be downloaded at: https://www.mdpi.com/article/10.3390/plants14071100/s1. Table S1: The 30 differential metabolites common to both 171 and 182. Table S2: The list of metabolites in Class3. Table S3: Differentially expressed genes (DEGs) list. Table S4: Ten predicted transcription factors and annotations within the 382 intersecting genes. Table S5: Correlation between metabolites and genes. Figure S1: KEGG pathway enrichment analysis to identify differentially abundant metabolites in the cold-tolerant okra variety Ae182 under chilling stress conditions. Figure S2: KEGG pathway enrichment analysis of differentially abundant metabolites in cold-tolerant okra Ae182. Figure S3: KEGG and GO enrichment analysis of differentially expressed genes.
