# Supplementary material for: Jasmonic Acid-Mediated Antioxidant Defense Confers Chilling Tolerance in Okra (Abelmoschus esculentus L.)
Source: Plants (Basel). 2025 Apr 2;14(7):1100. doi: 10.3390/plants14071100 (PMC11991441; doi:10.3390/plants14071100)
Supplement: Supplementary file 1 [file plants-14-01100-s001.zip › Supplementary Figure.pdf]

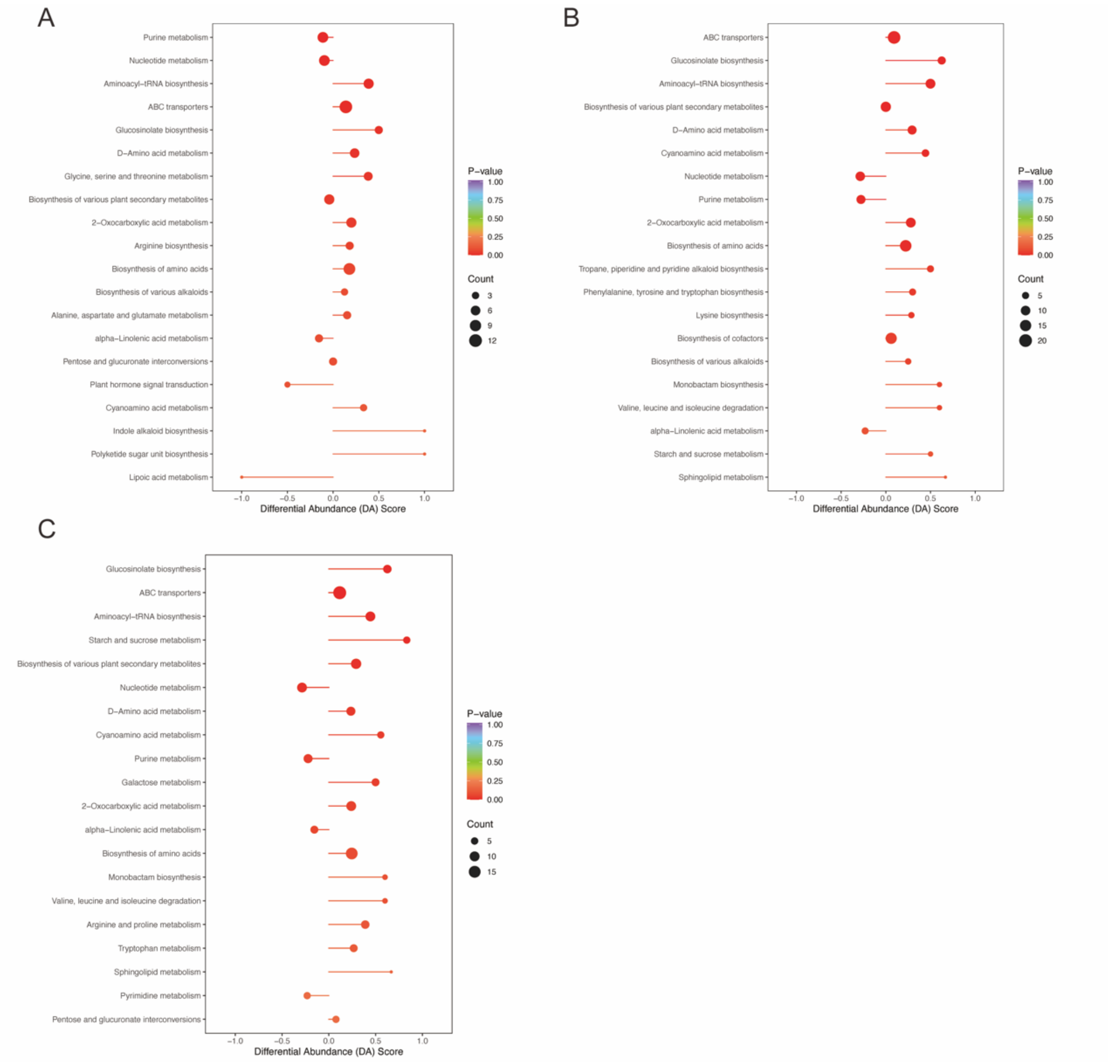

Supplementay Figure S1. KEGG pathway enrichment analysis was performed to identify differentially abundant metabolites in the cold-tolerant okra variety Ae182 under chilling stress conditions. (A) Comparison of Ae182-1d vs Ae182-0d. (B) Comparison of Ae182-3d vs Ae 182-0d. (C) Comparison of Ae182-5d vs Ae182-0d. Each point in the plot represents a specific metabolic pathway, with the x-axis denoting the differential abundance score (DA score). The color gradient reflects the enrichment p-value, indicating the statistical significance of the pathway enrichment, while the size of the circle corresponds to the number of differentially abundant metabolites associated with each pathway.

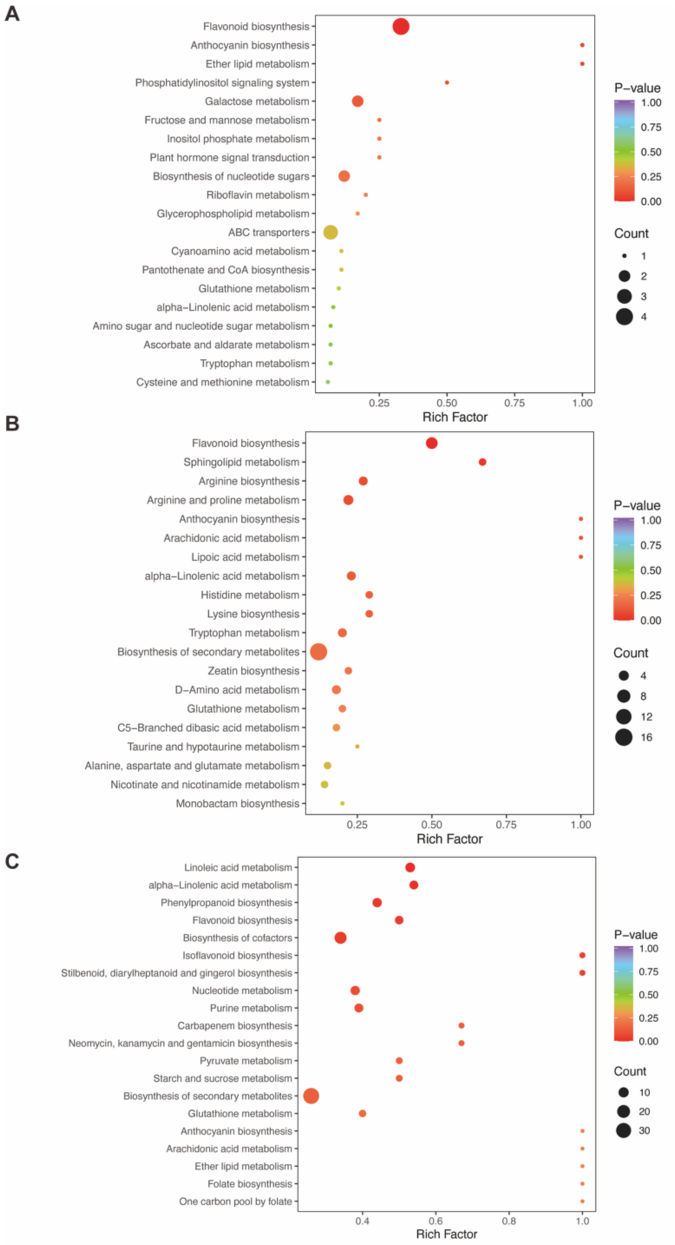

Supplementary Figure S2. KEGG pathway enrichment analysis of differentially abundant metabolites in cold-tolerant okra Ae182. (A) Comparison of metabolite profiles between Ae182 and Ae171 at 1 days. (B) Comparison between Ae182 and Ae171 at 3 days. (C) Comparison between Ae182 and Ae171 at 5 days. Each point represents a specific metabolic pathway, with the x-axis indicating the rich factor, reflecting the relative abundance of metabolites within each pathway. The color gradient represents the p-value of enrichment, with darker colors indicating more significant pathways, while the size of the circles corresponds to the number of differentially abundant metabolites associated with each pathway.

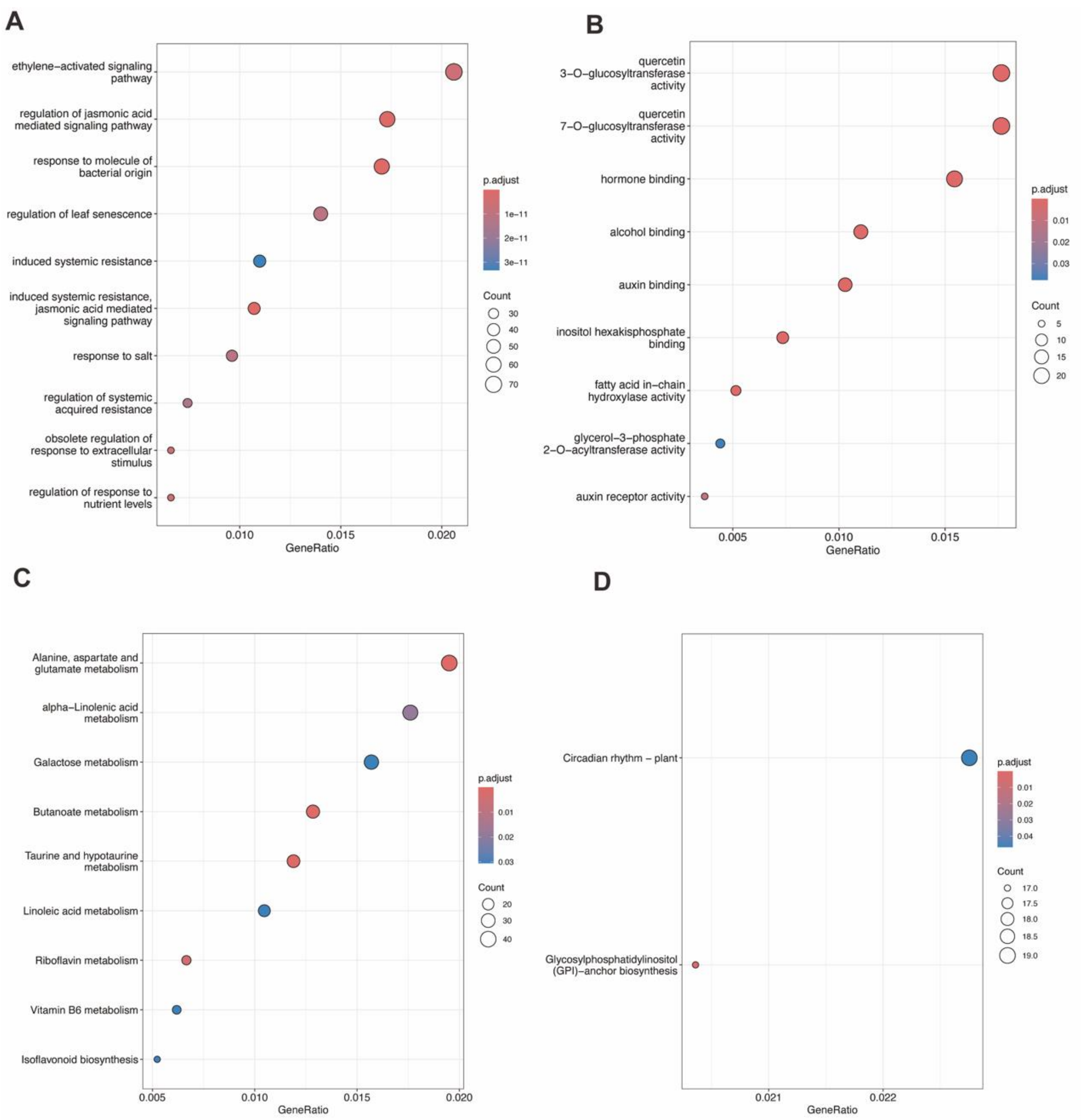

Supplementary Figure S3. KEGG and GO enrichment analysis of differentially expressed genes. (A) GO annotations for upregulated genes. (B) GO annotations for downregulated genes. (C) KEGG annotations for upregulated genes. (D) KEGG annotations for downregulated genes. Each point represents a specific metabolic pathway, with the x-axis indicating the rich factor, which reflects the relative abundance of genes within each pathway. The color gradient represents the p-value of enrichment, with darker colors indicating more significant pathways. The size of the circles corresponds to the number of differentially expressed genes associated with each pathway.
